# The Board of Health Resolutions

**Published:** 1879-05

**Authors:** 


THE BOARD OF HEALTH RESOLUTIONS.—The following resolutions have been passed by the Philadelphia Board of Health:—

*Resolved,* That hereafter, when death has resulted from a contagious disease, the Board of Health require that the cause shall be mentioned in the published death notice.

*Resolved,* That the public should avoid unnecessary attendance upon the funerals of those dead from contagious diseases.

*Resolved,* That among the diseases especially calling for this caution are, scarlet fever, measles, whooping cough, diphtheria, and smallpox or varioloid.

*Resolved,* That wherever possible, air-tight burial cases be used, and if such cannot be obtained, the funeral be strictly private.

